# Neurotoxic Alkaloids: Saxitoxin and Its Analogs

**DOI:** 10.3390/md8072185

**Published:** 2010-07-20

**Authors:** Maria Wiese, Paul M. D’Agostino, Troco K. Mihali, Michelle C. Moffitt, Brett A. Neilan

**Affiliations:** 1 School of Biotechnology and Biomolecular Sciences, University of New South Wales, Sydney, NSW, 2052, Australia; E-Mails: m.wiese@student.unsw.edu.au (M.W.); troco@unsw.edu.au (T.K.M.); 2 School of Biomedical and Health Sciences, University of Western Sydney, Campbelltown, NSW, 2560, Australia; E-Mails: p.dagostino@uws.edu.au (P.M.D.); m.moffitt@uws.edu.au (M.C.M.)

**Keywords:** saxitoxin, STX, paralytic shellfish poisoning, PSP, paralytic shellfish toxins, PSTs, neurotoxins, alkaloid analogs

## Abstract

Saxitoxin (STX) and its 57 analogs are a broad group of natural neurotoxic alkaloids, commonly known as the paralytic shellfish toxins (PSTs). PSTs are the causative agents of paralytic shellfish poisoning (PSP) and are mostly associated with marine dinoflagellates (eukaryotes) and freshwater cyanobacteria (prokaryotes), which form extensive blooms around the world. PST producing dinoflagellates belong to the genera *Alexandrium*, *Gymnodinium* and *Pyrodinium* whilst production has been identified in several cyanobacterial genera including *Anabaena*, *Cylindrospermopsis*, *Aphanizomenon Planktothrix* and *Lyngbya.* STX and its analogs can be structurally classified into several classes such as non-sulfated, mono-sulfated, di-sulfated, decarbamoylated and the recently discovered hydrophobic analogs—each with varying levels of toxicity. Biotransformation of the PSTs into other PST analogs has been identified within marine invertebrates, humans and bacteria. An improved understanding of PST transformation into less toxic analogs and degradation, both chemically or enzymatically, will be important for the development of methods for the detoxification of contaminated water supplies and of shellfish destined for consumption. Some PSTs also have demonstrated pharmaceutical potential as a long-term anesthetic in the treatment of anal fissures and for chronic tension-type headache. The recent elucidation of the saxitoxin biosynthetic gene cluster in cyanobacteria and the identification of new PST analogs will present opportunities to further explore the pharmaceutical potential of these intriguing alkaloids.

## 1. Introduction

The paralytic shellfish toxins (PSTs) are a group of naturally occurring neurotoxic alkaloids. Saxitoxin (STX) is the most researched PST to date, and since its discovery in 1957 [1], 57 analogs have been described. The PSTs are primarily produced in detrimental concentrations during harmful algal bloom (HAB) events [2–5] Over the last few decades, HABs have become more frequent, intense, and span a wider global distribution, the cause of which is still under debate [3,6]. The PSTs can be broadly characterized as hydrophilic or hydrophobic, and can be divided into subgroups based on substituent side chains such as carbamate, sulfate, hydroxyl, hydroxybenzoate, or acetate. Each moiety then imparts a varying level of toxicity [7].

In marine environments, PSTs are primarily produced by the eukaryotic dinoflagellates, belonging to the genera *Alexandrium*, *Gymnodinium* and *Pyrodinium* [8–10]. The toxins are passed through the marine food web via vector organisms, which accumulate the toxins by feeding on PST producing dinoflagellates without apparent harm to themselves [11,12]. These include filter feeding invertebrates such as shellfish, crustaceans, molluscs and also other, non-traditional vectors such as gastropods and planktivorous fish [13]. In freshwater environments the PSTs are produced by prokaryotic cyanobacteria belonging to the genera *Anabaena*, *Cylindrospermopsis*, *Aphanizomenon*, *Planktothrix* and *Lyngbya.* Cyanobacterial PST producing blooms result in the contamination of drinking and recreational water resources. In the past, high levels of toxins have been detected in the freshwater resources of many countries such as Australia, Brazil, USA, Mexico, Germany and China [14–22].

**Figure 1:**
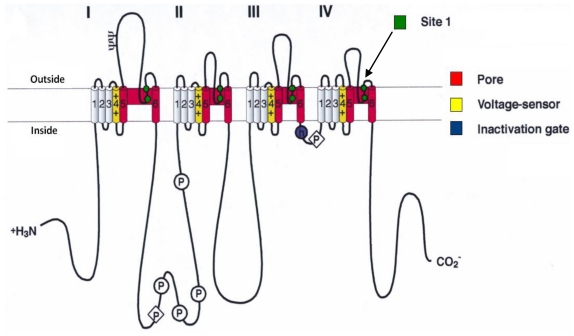
The proposed transmembrane arrangement of the α-subunit of Na^+^ channels. The pore is represented in red, the voltage sensors in yellow and the inactivation gate in blue. PSP is mediated by the interaction and blockage of Site 1 by STX. Figure adapted from [30].

Intoxication with PSTs may result in the severe and occasionally fatal illness known as paralytic shellfish poisoning (PSP) or saxitoxin pufferfish poisoning (SPFP) [23–27]. This illness is caused when PSTs reversibly bind voltage-gated Na^+^ channels in an equimolar ratio. This is mediated by the interaction between the positively charged guanidinium groups of STX with negatively charged carboxyl groups at site 1 of the Na^+^ channel, thereby blocking the pore (Figure 1) [28–30]. Currently, there is no antidote for PSP with artificial respiration and fluid therapy the only treatment available. A recent case of PSP involved the death of two fishermen after consumption of the filter feeder bi-valve *Aulacomya ater* in the Chilean Patagonian Fjords [26]. The threat of PSP is not only a major cause of concern for public health but is also detrimental to the economy. Outbreaks of PSTs often result in the death of marine life and livestock, the closure of contaminated fisheries, while the continual expenditure required for the maintenance and running of monitoring programs, all combine to present a major economic burden around the world [31,32].

This review will focus on the structural diversity of PSTs characterized to date and the biosynthetic and metabolic basis for this diversity. The saxitoxin biosynthetic gene cluster (*sxt*) was recently identified in cyanobacteria, which now provides insight into the biosynthesis of STX and its analogs [33,34]. A specific suite of analogs can be isolated from a single PST-producing organism, which is directly a result of the evolution of genes present within the organism’s genome [14,33–37]. Naturally occurring PSTs can also be precursors for extracellular metabolic or chemical transformations into new analogs. Knowledge of these transformations may have important implications for the detection, toxicity and removal of PSTs from a contaminated source. Other medicinal uses for PSTs may become more established by screening the bioactivity of less toxic analogs, since their use as a potential local anesthetic has long been known [38,39]. The characterization of PST biosynthesis genes and their potential use in combinatorial biosynthesis, together with the constant discovery of novel analogs (either natural or transformed), is likely to expand the possibilities for the pharmaceutical use of PSTs [40,41].

## 2. Saxitoxin and Its Analogs, the Paralytic Shellfish Toxins

STX is one of the most potent natural neurotoxins known. A dose of approximately 1 mg of the toxin from a single serving of contaminated shellfish is fatal to humans. STX was the first PST isolated in pure form from the Alaskan butter clam, *Saxidomus gigangteus* in 1957 [1]. Its highly polar characteristics represent poor conditions for crystallization and hampered structure elucidations for 18 years, until the crystal structure was solved by two groups independently in 1975 [42,43]. STX is an alkaloid with the molecular formula C_10_H_17_N_7_O_4_ (Molecular Weight = 299) and is composed of a 3,4-propinoperhydropurine tricyclic system. STX belongs to the large family of guanidinium-containing marine natural products, due to the presence of two guanidino groups which are responsible for its high polarity [44,45]. Since its initial discovery, 57 naturally occurring STX analogs have been identified in a number of organisms, collectively referred to as the PSTs (Table 1).

Usually a PST- producing organism synthesizes a characteristic suite of toxins made up of several PST analogs. These analogs differ in side group moieties and thus are commonly grouped according to these variable residues. The most commonly occurring PSTs are hydrophilic and have been studied in depth [7]. They may be non-sulfated, such as STX and neosaxitoxin (neoSTX), mono-sulfated, such as the gonyautoxins (GTXs 1–6), or di-sulfated (C1-4 toxins) [7,90]. In addition, decarbamoyl variants of these analogs also exist, including decarbamoyl-saxitoxins (dcSTX, dcneoSTX), decarbamoyl-gonyautoxins (dcGTXs 1–4), and the 13-deoxy-decarbamoyl derivatives (doSTX, doGTX 2,3). Three structural families of SXT are classified by the identity of the R_4_ side chain as either *N*-sulfocarbamoyl, decarbamoyl, or carbamoyl, each with increasing toxicity in mammalian bioassays (Table 2) [7,9,90]. Recently, an increase in screening efforts, coupled with improved methods for detection and structure elucidation, has seen an increase in the number of new PSTs reported in the literature.

A novel group of PSTs with a hydrophobic side chain were identified within the cyanobacterium *Lyngbya wollei* and are characterized by the presence of an acetate at C13 (LWTX 1–3,5,6) and a carbinol at C12 (LWTX 2,3,5) in place of a hydrated ketone [82]. This was the first report of STX derivatives with a hydrophobic substituent and these toxins have only been found exclusively in the freshwater environment [14,82]. The presence of an acetate side chain in the LWTXs correlated with a decrease in mouse toxicity, while the reduction at C12 resulted in a complete loss of mouse toxicity [82].

Interestingly, Negri *et al.* reported a novel subclass of analogs containing a hydrophobic R_4_ side chain designated GC1-3. These were first isolated and structurally characterized from Australian isolates of the dinoflagellate *Gymnodinium catenatum* and since have also been identified within *Alexandrium catenatum* globally [72]. High-resolution mass-spectrometry (MS) and nuclear magnetic resonance spectroscopy (NMR) revealed that GC3 is a 4-hydroxybenzoate ester derivative of dcSTX, while GC1 and GC2 are epimeric 11-hydroxysulfate derivatives of GC3 [83,91]. Negri *et al.* emphasized that the lipophilic nature of these toxins may lead to an increased potential to bioaccumulate in marine organisms [72]. These novel analogs have also been shown to bind strongly to the voltage gated Na^+^ channel. The binding affinity of GC3 resembles the affinity of the GTXs, whereas the epimer pair GC1 and GC2 bind with a similar affinity compared to the C-toxins [72,92]. More recently, other GC PST analogs have been identified, such as GC4-6, the di-hydroxylated benzoate GC analogs GC1-6a and the sulfated benzoate analogs GC1-6b for which only putative structures have been determined via mass spectrometry (MS) [85]. Due to their hydrophobic nature, these toxins easily escape conventional chromatography methods. The frequently used C18 solid-phase separation is based on polarity and thus hydrophobic compounds are retained on the column and cannot be detected. This is significant from a shellfish monitoring and public safety viewpoint, and presents a major challenge to water authorities [72,93,94].

Recently, Vale *et al.* reported the isolation of four unusual compounds (denoted A–D) and categorized them as novel STX analogs based on fluorescence emission, ultraviolet absorption maxima and cross-reactivity to a commercial antibody towards STX [86]. These extracts originated from shellfish samples (*Semele proficua* and *Senilia senilis*) collected from Luanda and Mussulo Bay, Angola. Compounds A and D were classified as non-N1-hydroxyl PST analogs and compound B as a N1-hydroxyl analog. Even though the presence of *G. catenatum* and *Pyrodinium bahamense* has been reported from the coast of Angola, none of the 18 PSTs commonly found in dinoflagellates were identified in these extracts. The authors therefore suggested a possible cyanobacterial source, though neither a definitive chemical structure, nor a PST-producing organism were conclusively identified [86]. Further analysis of the compounds by MS and NMR is required to elucidate these structures and confirm them as STX analogs.

The most exotic STX isolate identified to date was isolated from the Panamanian golden frog *Atelopus zeteki* and designated zetekitoxin AB (Tables 1 and 2). Zetekitoxin AB was confirmed to be a PST containing a unique 1,2-oxazolidine ring-fused lactam. The binding affinity of zetekitoxin AB for brain, heart, and muscle Na^+^ channels was extremely potent, displaying a toxicity of approximately 580-, 160- and 63-fold greater than STX against each channel, respectively [89].

The constant discovery of novel and diverse STX analogs is a challenge to PST identification and monitoring. Improvement of detection methods will no doubt uncover new natural forms of STX, however, we are still only beginning to understand the mechanisms by which these complex molecules are produced in nature.

## 3. Biotransformation of the Paralytic Shellfish Toxins

**Figure 2:**
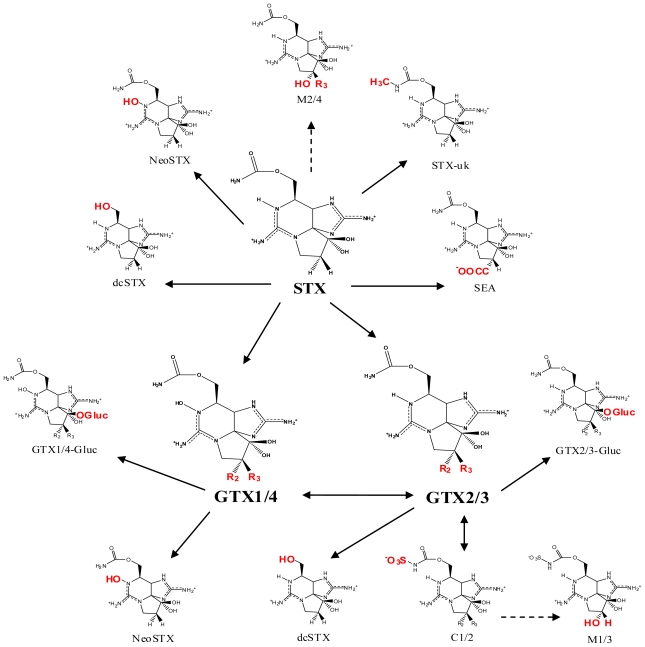
Biotransformation of the paralytic shellfish toxins. Refer to Table 1 for assigned R groups. Moieties highlighted in red indicate a differentiation from the structure of STX. Unbroken line refers to experimental data of toxin conversion. Broken line refers to putative biotransformation based on structural analysis.

Naturally occurring PSTs may be structurally modified by various biological factors. In some cases, these biotransformations can result in new PSTs that cannot be biosynthesized by cyanobacteria or dinoflagellates alone (Figure 2). In addition, less toxic PSTs may be converted into analogs with greater toxicity (e.g., C-toxins→GTXs) or *vice versa*. Therefore, a clearer understanding of PST biotransformation is needed for predicting more accurate levels of toxicity. This knowledge may also allow for a mechanism of detoxification to be established and utilized in the water supply and shellfish farming industries.

Cell extracts of PST-producing dinoflagellates are capable of enzymatically modifying PSTs. Oshima *et al.* demonstrated that GTX2 + 3 can be converted into GTX1 + 4 by incubation with *Alexandrium tamarense* homogenate [92]. Introduction of a sulfate moiety on the carbamoyl group, resultingin the formation of C1 and C2 toxins, has been shown following incubation with *G. catenatum* homogenate [44,99]. In these organisms, biotransformation is likely to occur via inherent STX tailoring enzymes which are a part of the SXT biosynthetic pathway encoded within the organism.

Due to differences in the toxin profiles of filter-feeding invertebrate PST vectors and causative producing organisms, various studies have been conducted to monitor toxin biotransformation [84,100–105]. Enzymatic transformation of carbamoyl and carbamoyl-*N* sulfated toxins into the decarbamoyl compounds was detected within the little neck clam, *Prothotheca staminea* [106]. In addition, the conversion of the GTXs and neoSTX to STX by reduction of the O22-sulfate and N1-hydroxyl groups, respectively, has been observed within the homogenate of the scallop *Placopecten magellanicus* [107].

GC1-3 can be converted into dcSTX, as has been confirmed *in vitro* through incubation of semi-purified GC toxins with bivalve digestive glands [93]. Similarly, the recently identified M-toxins (M1-5) are reportedly bivalve metabolites of the PSTs and are not present in PST- producing microalgae [56]. The M-toxins constitute an important toxin fraction in mussels contaminated by *A. tamarense* and *G. catenatum* and have been detected in shellfish, including mussels, cockles and clams [56,86]. These findings are similar to previous reports on the isolation of 11-saxitoxinethanoic acid (SEA), a novel PST from the xanthid crab *Atergatis floridus*, inhabiting the pacific coast of Shikoku Island [87]. Other examples include a novel carbamoyl-*N-*methylsaxitoxin (STX-uk) isolated from the Bangladeshi freshwater puffer *Tetraodon cutcutia* [88]. These exotic STX analogs are likely products of toxin transforming enzymes within the vector organism or its associated microorganisms. However, the mechanism of enzymatic transformation in these organisms is yet to be elucidated [56,86–88,106–109].

Biotransformation of the PSTs by bacteria was first suggested many years ago by Kotaki *et al.*, who proposed that marine bacteria, such as *Vibrio* and *Pseudomonas* spp., are capable of metabolizing PSTs [110]. In addition, isolates from the viscera of marine crabs, snails and the marine red algae *Jania* sp., were studied and demonstrated transformation GTX derivatives into STX through reductive eliminations [110,111]. Bacterial conversion of GTX1-4 to STX and neoSTX is reportedly due to the bacterial thiol compounds glutathione and 2-mercaptoethanol [112]. The ability of bacteria to degrade PSTs has been further described by Smith *et al.*, who screened marine bacterial isolates from various shellfish species for their ability to metabolize a range of PSTs, such as GTX1-5, STX and neoSTX, suggesting that bacteria might play an important role in the clearance of PSTs from bivalve molluscs [113]. Novel strains of *Pseudoalteromonas haloplanktis*, isolated from the digestive tracts of blue mussels (*Mytilus edulis*) have been reported to possess the ability to reduce the overall toxicity of a PST mixture of algal extracts by 90% within three days [114,115]. Catabolism of the PSTs most likely occurred via oxidation reactions catalyzed by oxidases and peroxidases into aliphatic products for subsequent use in purine and arginine metabolism, although this is speculated, as no catabolized PST products could be identified [115]. Degradation has also been observed during the passage through a bioactive treatment plant, leading to a decrease in predominant C-toxins and an increase of GTX2 + 3 which display relatively higher toxicity [116].

### Detoxification of the paralytic shellfish toxins within mammals

Metabolism of PSTs by humans has not been studied in depth. Nevertheless, Garcia *et al.* suggested biotransformation of STX to neoSTX and the oxidation of the GTX2 + 3 epimers into GTX1 + 4 within samples of pancreas, bile, urine, brain and heart obtained post-mortem from PSP victims [26]. Further investigations confirmed their findings of biotransformation in humans. N1-oxidation of GTX2 + 3 into the corresponding hydroxylamine analogs GTX1 + 4 has been demonstrated *in vitro* when incubated with a microsomal fraction isolated from healthy human livers. Moreover, *in vitro* glucuronidation of GTX2 + 3 into the hydrophilic compounds GTX3-Gluc and GTX2-Gluc, through conjugation at the hydroxyl-C12 group has also been reported (Figure 2) [117]. The oxidation and glucuronidation of STX and GTX2 + 3 epimers into neoSTX or GTX1 + 4 epimers, respectively, has been suggested to be significant detoxification pathways of GTX2 + 3 and other PSTs in humans and other mammals [117]. Similar studies were conducted with cat liver, however, enzymatic transformation was not detected, with 100% recovery of the STX used in the incubation being recovered [118]. This was explained by the fact that with the exception of cats, the liver of mammals produces glucuronides as a major metabolic product, thus supporting the specificity of human tissue transformation [119]. However, biotransformation of STX was not detected when STX was passaged through rat’s urine, indicating further mammalian variability in models [120,121]. Gessner *et al.* investigated serum and urine in human PSP victims and detected a significant increase of the PST C1 in comparison to GTX2, which is distinguished by an additional sulfate on the carbamoyl side group [122]. A new assay for STX and neoSTX quantification in human urine samples has been developed recently [123]. It is proposed that methodological improvements should also contribute to a better understanding of PST profile and its change while passaging through the human body [123].

The research described above highlights the need to characterize the diversity of biological transformations of PSTs. Detoxification pathways could be manipulated to improve biological removal strategies, while further characterization of detoxification of PSTs within the human body could lead to improved treatment of PSP.

## 4. A Genetic Basis for the Paralytic Shellfish Toxins

### 4.1. The saxitoxin biosynthetic gene cluster

Recently the saxitoxin biosynthesis pathway was proposed [124], and the s*xt* gene cluster was identified in three cyanobacterial species of the family *Nostocaceae* [33,34] and one from the family *Oscillatoriaceae* [125]. The *sxt* gene clusters within each organism all contain a core set of genes putatively responsible for the biosynthesis of STX. However, the gene profile between each cluster differs, resulting in the production of a different suite of STX analogs by each organism. It is foreseeable that identification of the cyanobacterial PST biosynthesis genes will eventually lead to the identification of the homologs within dinoflagellates. However, the dinoflagellate PST biosynthesis genes remain elusive. There is also some debate on whether the enzymes for PST biosynthesis are encoded by the dinoflagellate genome, including plastids or other sources such as symbiotic bacteria or viruses [126–128].

In cyanobacteria, biosynthesis of STX is catalyzed by several enzymes otherwise rare in microbial metabolism. The core PST biosynthetic gene, *sxtA*, is thought to have a chimeric origin and is putatively responsible for the initiation of STX biosynthesis, catalysing the incorporation of acetate to the enzyme complex and its subsequent methylation and Claisen condensation with arginine [33,34,129]. SxtA consists of four catalytic domains (SxtA1-SxtA4) with the *N*-terminal region showing similarities to a polyketide synthase (PKS) complex [130] consisting of a GCN5-related *N*-acetyltransferase [131], acyl-carrier protein (ACP) and a S-adenosylmethionine-dependant (SAM) methyltransferase [132] domains, while the *C*-terminal region contains a domain homologous to previously characterized aminotransferases [133].

Specific PST analog profiles are proposed to be the result of tailoring enzymes encoded by the *sxt* gene cluster. The function of tailoring enzymes within each of the characterized *sxt* clusters has been inferred by analysis of the specific toxin profile produced by each cyanobacterium. For example, neoSTX differs from STX by hydroxylation at the N1 position (Table 1). NeoSTX is produced by *C. raciborskii* T3, *Aphanizomenon* sp. NH-5 and *L. wollei*, but has not been detected in *A. circinalis* [14,35,36,57,62]. Sequence analysis of the four *sxt* gene clusters revealed SxtX as a protein putatively responsible for the N1-hydroxylation of STX, since *sxtX* was identified in all neoSTX producing strains and absent from the *A. circinalis* AWQC131C gene cluster [33,34]. This protein displayed high structural similarities to cephalosporin hydroxylase [134], further affirming its role in the *N*1-hydroxylation of STX.

The GTXs are produced by mono-sulfation at N21 or O22 of STX which can then be di-sulfated to produce the C-toxins. Previous studies of the dinoflagellate *G. catenatum*, revealed two 3′-phosphate 5′-phosphosulfate (PAPS)-dependant sulfotransferases responsible for the N21 sulfation of STX, GTX2 and GTX3, and the O22 sulfation of 11-hydroxy STX [135,136]. Two genes, *sxtO*, a PAPS forming enzyme and *sxtN*, a sulfotransferase, within cyanobacterial *sxt* clusters are proposed to encode proteins that play a similar sulfation role in the synthesis of GTXs and C-toxins.

The requirement of SAM for STX biosynthesis has long been hypothesized and thus has been targeted during attempts to identify the PST genes [137,138]. Harlow *et al.* were able to use degenerate primers to screen several dinoflagellate genomes in an attempt to identify genes encoding SAM as a candidate involved in PST biosynthesis [138]. Although several SAM genes were successfully identified within dinoflagellates, these were not correlated to PST biosynthesis. The study was hampered by a limited knowledge of dinoflagellate codon usage and a lack of related sequence information within the NCBI database [138,139]. Kellmann *et al.* used a similar degenerate PCR approach to identify a gene encoding a *O*-carbamoyltransferase (*sxtI*), which ultimately led to the identification of the entire *sxt* biosynthesis pathway in cyanobacteria [33,138,140]. There are now multiple genes that may be utilized to target homologs of the *sxt* cluster in dinoflagellates. However, a recent study identified the dinoflagellate *sxt* cluster may differ from cyanobacteria more than would be expected from a recent gene transfer event. Hence, mRNA present solely within toxic dinoflagellates may be more successful at identifying the candidate *sxt* pathway in these organisms [141].

### 4.2. Pharmaceutical potential of the paralytic shellfish toxins

Recent years has seen a renewed interest in marine alkaloids and their analogs, including the PSTs, with regards to their use as therapeutic agents or as a drug lead. Bioactivity studies and molecular modeling of a range of PSTs could also lead to the design of unnatural analogs with improved pharmaceutical characteristics. Recently, a group of toxins isolated from marine cone snails (genus *Conus*), known as conotoxins, have been shown to contain over 2,000 peptide analogs [142]. The conotoxins are able to specifically target a broad range of ion channels and membrane receptors with several currently under investigation for possible clinical trials [142]. In 2004, a synthetic version of a single conotoxin analog, ω-conotoxin MVIIA, also known as ziconotide (trade name Prialt^®^) was the first marine natural product to be approved for use by the US Food and Drug Administration since 1976 [143,144]. Ziconotide acts by targeting N-type voltage sensitive Ca^2+^ channels and is used for the treatment of chronic pain in spinal cord injury [145,146].

Like Prialt^®^, STX also has a huge pharmaceutical potential for its ability to induce anesthesia through interaction with site 1 of the voltage gated Na^+^ channel [38,39]. It has been suggested that site 1 blockers prolong the duration of anaesthesia in a synergistic manner when combined with other local anaesthetics [39,147,148]. In spite of this, the push for STX to enter clinical trials has been hindered by its systematic toxicity [149]. The use of STX as a slow release, prolonged anesthetic was recently demonstrated using a novel controlled release system in male Sprague-Dawley rats [150]. Liposomal formulations of STX, either alone and in conjunction with dexamethasone and/or bupivacaine, were able to block the sciatic nerve within rats for long periods with no damaging myotoxic, cytotoxic or neurotoxic effects and little associated inflammation [150]. Liposome formulations of STX for slow and site-directed release for prolonged anaesthesia have since been postulated as a putative treatment of localized pain and severe joint pain [151].

PSTs such as GTX2 + 3 also have clinical potential and have been utilized for the treatment of anal fissures [152–154]. Since 1951, surgery has been the most common form of anal fissure treatment with several possible side effects [155–157], while other treatments include ointments [158], botulinium toxin [159] and topical application of nitroglycerine [160]. Treatment with GTX2 + 3 involves direct injection into both sides of the fissure. A success rate of 98% with remission after 15 and 28 days for acute and chronic conditions, respectively (*n* = 100) was observed [153]. A follow up study with an enhanced method has since been performed by Garrido *et al*. with an improved time of healing of seven to 14 days for chronic cases (*n* = 23) [154]. Both studies identified GTX2 + 3 as safe and effective when compared to other treatments [153,154]. GTX2 + 3 have also been used in the treatment of chronic tension type headache, with 70% of patients (*n* = 27) responding to treatment [161]. These studies recognize that PSTs other than STX also have potential as future pharmaceutical leads. Their use in the past has also been limited largely due to problems obtaining purified PST analogs.

The genetic characterization of PST biosynthesis pathways from diverse producer organisms has increased our insight into *sxt* tailoring reactions and the molecular understanding of the mechanisms by which a particular suite of PSTs can be synthesized. This will ultimately advance research into the pharmaceutical potential of the PSTs as Na^+^ channel blockers, by generating new analogs or by increasing the availability of analogs otherwise biosynthesized in low concentrations. Bioengineering can also be utilized to further enhance the structural diversity of bioactive small molecules by using *in vitro* approaches that utilize enzymes in chemical synthesis, as well as *in vivo* approaches, such as combinatorial biosynthesis [40,41]. Combinatorial biosynthesis is the process of incorporating genes from multiple biosynthetic clusters into an expression plasmid, in a combinatorial fashion, to generate a library of “unnatural” natural products expressed *in vivo*. However expression of large gene fragments in a heterologous host is required and analogs of interest may then be extracted, purified and assayed to determine their bioactivity.

The bioactive nature of STX as an anaesthetic and GTX2 + 3 for the treatment of anal fissures and chronic tension type headaches demonstrates that these alkaloids have pharmaceutical potential deserving of further investigation. The recent elucidation of the *sxt* gene clusters in cyanobacteria and the identification of novel PSTs has provided more options for further PST bioactivity studies. Novel analogs could also be devised by redesigning PST biosynthesis genes in amenable host systems via combinatorial biosynthesis.

## 5. Conclusions

The structure of STX has been known for 53 years and the discovery of novel STX analogs has continued steadily ever since. Today, 57 PST analogs have been reported. With more sensitive detection methods, new STX analogs will most likely continue to be identified, with new functional moieties and possibly novel bioactivity. Despite extended research on the role of saxitoxin and its analogs as a sodium channel blocker, the effect of these toxins on the environment, and the genes that are responsible for their production, there is still a vast gap in knowledge in regards to their potential intracellular role within the producing organism. Nevertheless, it is possible that the different analogs display varying functions within the cells due to their partial differences in charges and chemical properties. More studies are needed to elucidate the localization of saxitoxin and its derivatives might provide clues to the potential role of the PST analogs within the producing organism. In the future, a better understanding of the intracellular and extracellular functions of STX might open more avenues for pharmaceutical applications.

Since PSTs are produced by distantly related organisms, spanning two domains, including cyanobacteria, dinoflagellates and the Panamanian golden frog, it is possible that their occurrence in nature is more widespread than we know. Further investigations are needed to elucidate the extent of their distribution, diversity and their fundamental biology, such as their biosynthesis, metabolic and eco-physiological function. This is in addition to the role of chemical transformation of the different toxins in shellfish and the environment.

Future research is also needed to understand the integration of PST biosynthesis within the overall cell metabolism and the possible recruitment of enzymes from other biosynthetic pathways for PST bioconversions. Proteomic and transcriptomic studies are likely to provide a link between STX biosynthesis, regulation and cellular metabolism. It is expected that data will allow us to acquire a better understanding of the conservation of the SXT biosynthesis pathway at the enzymatic level in comparison to the genetic level, may give further insight into the molecular function of these toxins and also lead to clues of their evolutionary history. In future, characterization of PST biosynthetic genes from dinoflagellates and comparison with cyanobacterial genes will also aid in our understanding of the evolutionary history of these genes with regard to their origin and transfer.

PSP is a serious health problem and its incidence has continued to rise on a global scale. PSTs negatively impact the fisheries industry globally and the development of novel methods of detoxification is essential from a human health and financial perspective [104,113,162]. The enzymatic basis for the structural diversity of PSTs is now beginning to be understood from the genetics of their biosynthesis in cyanobacteria and characterization of transformations catalyzed by bacteria, marine invertebrates and mammals. Biotransformation pathways could also be manipulated to efficiently remove toxins from water supplies. Specific enzymes or bacterial strains that degrade PSTs could be introduced into shellfish to assist detoxification. Currently, the PSTs represent extraordinary potential for pharmacy. This potential is likely to increase as we continue to gain a better molecular understanding of the PSTs, leading to future prospects of their use in combinatorial biosynthesis for the production of novel alkaloids with beneficial application.

## Figures and Tables

**Table 1:** The paralytic shellfish toxins.

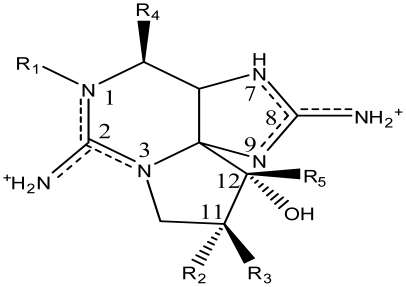							
Toxin	R1	R2	R3	Ω R4	R5	Origin	Ref.
STX	H	H	H	OCONH_2_	OH	*Alexandrium andersoni*	[46]
						*A. catenella*	[47–49]
						*A. fundyense*	[50–52]
						*A. tamarense*	[53–56]
						*A. circinalis*	[35,57–59]
						*Aphanizomenon flos-aquae*	[60–63]
						*Aph. gracile*	[20,64]
						*Aph. issatschenkoi*	[65]
						*Anabaena lemmermannii*	[66]
						*C. raciborskii*	[16,36,67–69]
						*Gymnodinium catenatum*	[70–72]
						*Pyrodinium bahamense*	[10]
						*Planktothrix* sp.	[73]
neoSTX	OH	H	H	OCONH_2_	OH	*A. andersoni*	[46]
						*A. catenella*	[47–49]
						*A. fundyense*	[50–52]
						*A. tamarense*	[53–56]
						*Aph. flos-aquae*	[60–63]
						*Aph. gracile*	[20,64]
						*Aph. issatschenkoi*	[65]
						*Aph.* sp.	[74]
						*C. raciborskii*	[16,36,69]
						*G. catenatum*	[70,71]
						*P. bahamense*	[10]

**Mono-Sulfated**							
GTX1	OH	H	OSO_3_^−^	OCONH_2_	OH	*A. catenella*	[47–49,75,76]
						*A. fundyense*	[50–52]
						*A. minutum*	[77–79]
						*A. tamarense*	[53–56]
						*Aph. flos-aquae*	[37]
						*G. catenatum*	[9,70,72]
GTX2	H	H	OSO_3_^−^	OCONH_2_	OH	*A. catenella*	[48,49]
						*A. fundyense*	[50–52]
						*A. minutum*	[77–79]
						*A. ostenfeldii*	[80]
						*A. tamarense*	[53–56]
						*A. circinalis*	[35,57–59]
						*C. raciborskii*	[36,67]
						*G. catenatum*	[9,70,72]
GTX3	H	OSO_3_^−^	H	OCONH_2_	OH	*A. catenella*	47–49]
						*A. fundyense*	[50–52]
						*A. minutum*	[77–79]
						*A. ostenfeldii*	[80]
						*A. tamarense*	[53–56]
						*A. circinalis*	[35,57–59]
						*Aph. flos-aquae*	[37]
						*C. raciborskii*	[36,67]
						*G. catenatum*	[9,70,72]
GTX4	OH	OSO_3_^−^	H	OCONH_2_	OH	*A. catenella*	[47–49,75,76]
						*A. fundyense*	[50–52]
						*A. minutum*	[77–79]
						*A. tamarense*	[53–56]
						*Aph. flos-aquae*	[37]
						*G. catenatum*	[9,70,72]
GTX5 (B1)	H	H	H	OCONHSO_3_^−^	OH	*A. catenella*	[48,49,75,76]
						*A. fundyense*	[50–52]
						*A. tamarense*	[54,56]
						*A. circinalis*	[35,57,59]
						*Aph. flos-aquae*	[60,63]
						*Aph. gracile*	[20]
						*Aph. issatschenkoi*	[37,65]
						*G. catenatum*	[9,71,81]
						*P. bahamense*	[10]
GTX6 (B2)	OH	H	H	OCONHSO_3_^−^	OH	*A. catenella*	[47,49,75,76]
						*A. fundyense*	[52]
						*A. ostenfeldii*	[80]
						*A. tamarense*	[54]
						*Aph. flos-aquae*	[63]
						*C. raciborskii*	[69]
						*G. catenatum*	[9,71,72,81]
						*P. bahamense*	[10]

**Di-Sulfated**							
C1	H	H	OSO_3_^−^	OCONHSO_3_^−^	OH	*A. catenella*	[48,49,75,76]
						*A. fundyense*	[50–52]
						*A. ostenfeldii*	[80]
						*A. tamarense*	[53–56]
						*A. circinalis*	[35,57–59]
						*C. raciborskii*	[68]
						*G. catenatum*	[9,71,72,81]
C2	H	OSO_3_^−^	H	OCONHSO_3_^−^	OH	*A. catenella*	[48,49,75]
						*A. fundyense*	[50–52]
						*A. ostenfeldii*	[80]
						*A. tamarense*	[53–56]
						*A. circinalis*	[35,57–59]
						*C. raciborskii*	[68]
						*G. catenatum*	[9,71,72,81]
C3	OH	H	OSO_3_^−^	OCONHSO_3_^−^	OH	*A. catenella*	[48,49,75,76]
						*G. catenatum*	[9,72,81]
C4	OH	OSO_3_^−^	H	OCONHSO_3_^−^	OH	*A. catenella*	[48,49,75,76]
						*G. catenatum*	[9,72,81]

**Decarbamoylated**							
dcSTX	H	H	H	OH	OH	*A. catenella*	[49]
						*A. circinalis*	[35,59]
						*Aph. flos-aquae*	[60,63]
						*Aph. gracile*	[20]
						*Aph. issatschenkoi*	[65]
						*Aph.* sp.	[74]
						*C. raciborskii*	[16,67,69]
						*Lyngbya wollei*	[82]
						*G. catenatum*	[9,71,72]
						*P. bahamense*	[10]
dcneoSTX	OH	H	H	OH	OH	*C. raciborskii*	[69]
dcGTX1	OH	H	OSO_3_^−^	OH	OH	*G. catenatum*	[83]
dcGTX2	H	H	OSO_3_^−^	OH	OH	*A. catenella*	[49]
						*A. fundyense*	[52]
						*A. circinalis*	[35,57–59]
						*G. catenatum*	[9,71]
						*L. wollei*	[14,82]
dcGTX3	H	OSO_3_^−^	H	OH	OH	*A. catenella*	[49]
						*A. fundyense*	[50,52]
						*A. circinalis*	[35,57–59]
						*Aphanizomenon* sp.	[74]
						*L. wollei*	[14,82]
						*G. catenatum*	[9,71]
dcGTX4	OH	OSO_3_^−^	H	OH	OH	*G. catenatum*	[83]

**Deoxy-Decarbomoylated**							
doSTX	H	H	H	H	OH	*G. catenatum*	[9,84]
doGTX1	OH	H	OSO_3_^−^	H	OH	*G. catenatum*	[9,84]
doGTX2	H	H	OSO_3_^−^	H	OH	*G. catenatum*	[9,84]

***L. wollei*toxins**							
LWTX1	H	H	OSO_3_^−^	OCOCH_3_	H	*L. wollei*	[82]
LWTX2	H	H	OSO_3_^−^	OCOCH_3_	OH	*L. wollei*	[82]
LWTX3	H	OSO_3_^−^	H	OCOCH_3_	OH	*L. wollei*	[82]
LWTX4	H	H	H	H	H	*L. wollei*	[82]
LWTX5	H	H	H	OCOCH_3_	OH	*L. wollei*	[82]
LWTX6	H	H	H	OCOCH_3_	H	*L. wollei*	[82]

**Mono-Hydroxy-Benzoate Analogs**							
GC1	H	H	OSO_3_^−^	OCOPhOH	OH	*G. catenatum*	[83]
GC2	H	OSO_3_^−^	H	OCOPhOH	OH	*G. catenatum*	[83]
GC3	H	H	H	OCOPhOH	OH	*G. catenatum*	[83]
*GC4	OH	H	OSO_3_^−^	OCOPhOH	OH	*G. catenatum*	[85]
*GC5	OH	OSO_3_^−^	H	OCOPhOH	OH	*G. catenatum*	[85]
*GC6	OH	H	H	OCOPhOH	OH	*G. catenatum*	[85]

**Di-Hydroxy Benzoate Analogs**							
ŧGC1a	H	H	OSO_3_^−^	DHB	OH	*G. catenatum*	[85]
ŧGC2a	H	OSO_3_^−^	H	DHB	OH	*G. catenatum*	[85]
ŧGC3a	H	H	H	DHB	OH	*G. catenatum*	[85]
ŧGC4a	OH	H	OSO_3_^−^	DHB	OH	*G. catenatum*	[85]
ŧGC5a	OH	OSO_3_^−^	H	DHB	OH	*G. catenatum*	[85]
ŧGC6a	OH	H	H	DHB	OH	*G. catenatum*	[85]

**Sulfated Benzoate Analogs**							
ŧGC1b	H	H	OSO_3_^−^	SB	OH	*G. catenatum*	[85]
ŧGC2b	H	OSO_3_^−^	H	SB	OH	*G. catenatum*	[85]
ŧGC3b	H	H	H	SB	OH	*G. catenatum*	[85]
ŧGC4b	OH	H	OSO_3_^−^	SB	OH	*G. catenatum*	[85]
ŧGC5b	OH	OSO_3_^−^	H	SB	OH	*G. catenatum*	[85]
ŧGC6b	OH	H	H	SB	OH	*G. catenatum*	[85]

**Other PST Analogs**							
M1	H	OH	H	OCONHSO_3_^−^	OH	Metabolic transformation	[56,81]
M2	H	OH	H	OCONH_2_	OH	Metabolic transformation	[56]
M3	H	OH	OH	OCONHSO_3_^−^	OH	Metabolic transformation	[56]
M4	H	OH	OH	OCONH_2_	OH	Metabolic transformation	[56]
*M5						Metabolic transformation	[56]
*A						Unknown	[86]
*B						Unknown	[86]
*C						Unknown	[86]
*D						Unknown	[86]
SEA	H	CCOO^−^	H	OCONH_2_	OH	*Atergatis floridus*	[87]
STX-uk	H	H	H	OCONHCH_3_	OH	*Tetraodon cutcutia*	[88]
Zetekitoxin AB						*Atelopus zeteki*	[89]
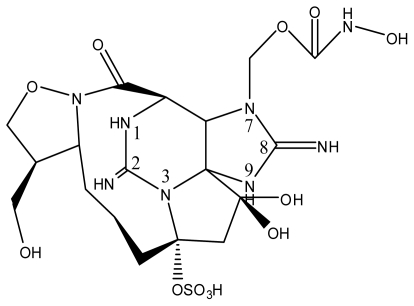							

* Not structurally characterized

ŧ R_4_ group putatively assigned based on major ions obtained via MS [85]

Ω OCONH_2_ 
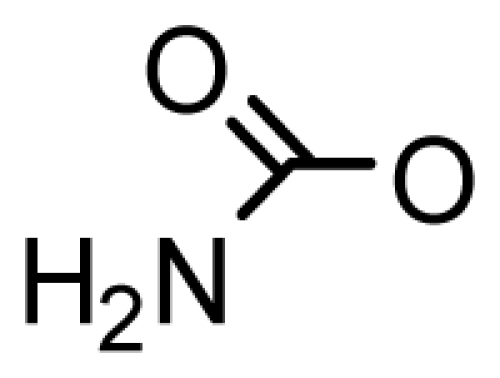

Ω OCONHSO_3_^−^ 
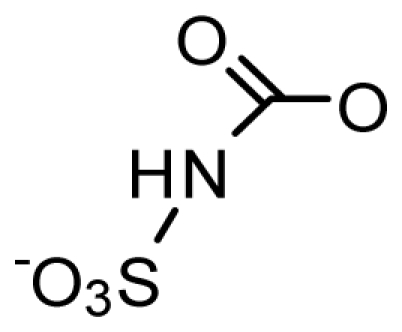

Ω OCOCH_3_ 
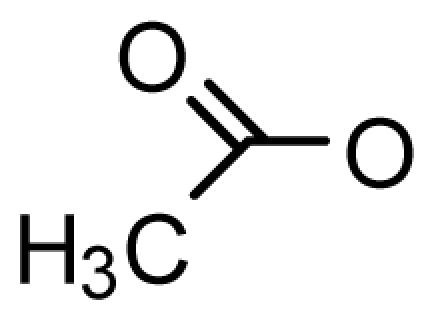

Ω OCOPhOH 
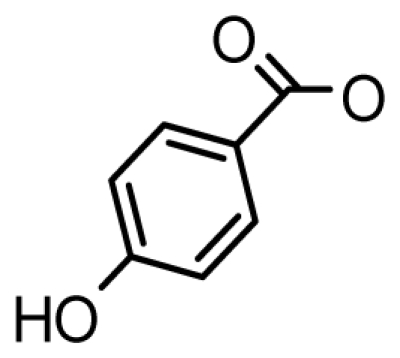

Ω OCONHCH_3_ 
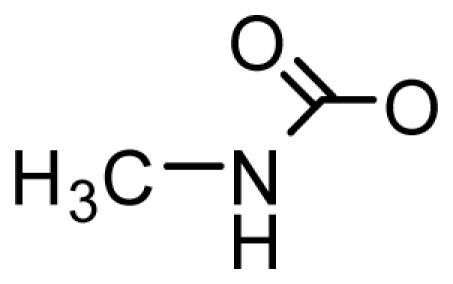

Ω DHB: Di-hydroxyl-benzoate

Ω SB: Sulfated-benzoate

**Table 2:** Relative toxicity of the paralytic shellfish toxins. Toxicity of the PSTs due to change in moiety is listed in descending order. Data obtained from [95].

StructureΩ	Toxin	Relative toxicityΦ
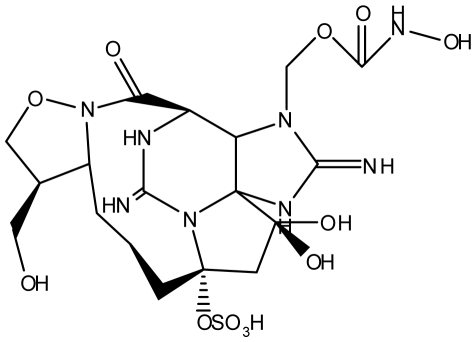	Zetekitoxin AB	63, 160, 580ω

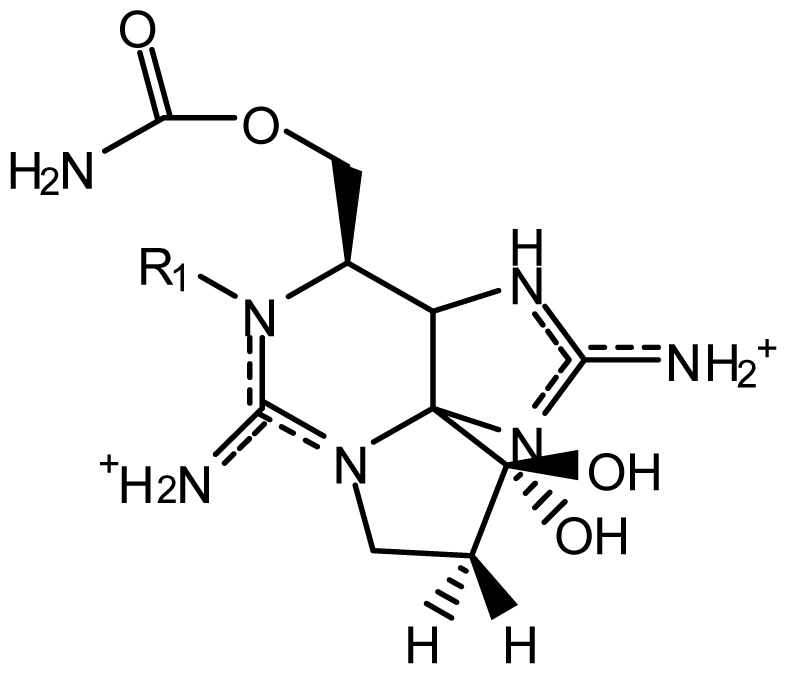	**Non-Sulfated**	
	STX NeoSTX	1 05–1.1

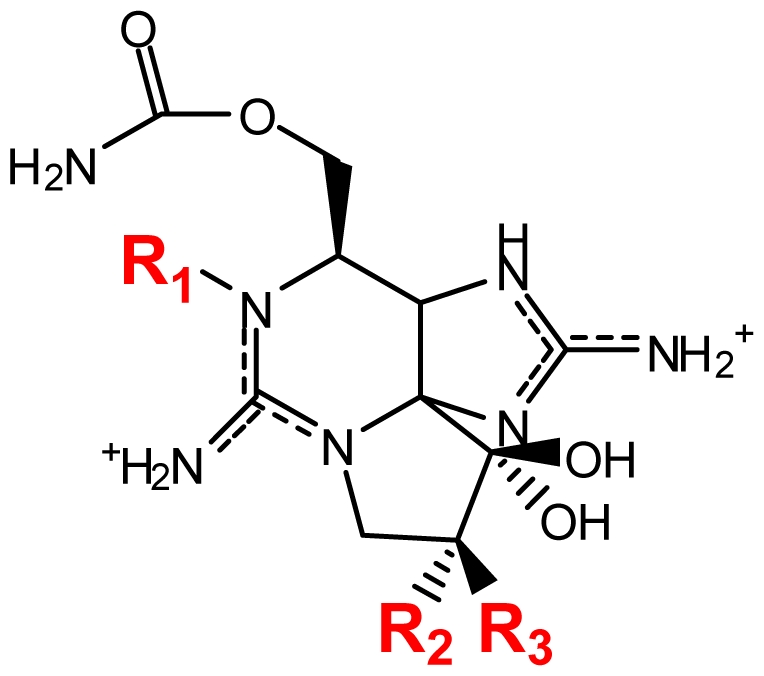	**Mono-sulfated**	
	GTX1/4¥ GTX2/3¥	0.39/1.09–0.48/0.76 0.8/0.33–0.9/0.9

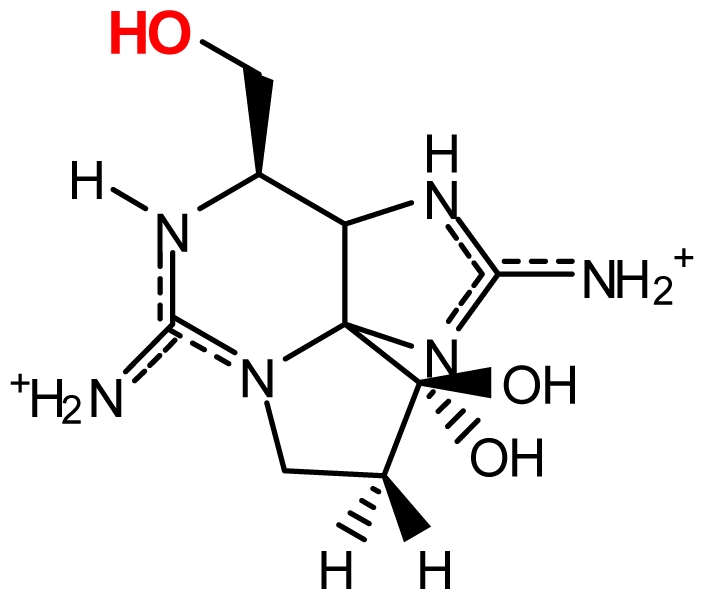	**Decarbamoylated**	
	dcSTX dcNeoSTX dcGTX1-4	0.43 0.43 0.18–0.45

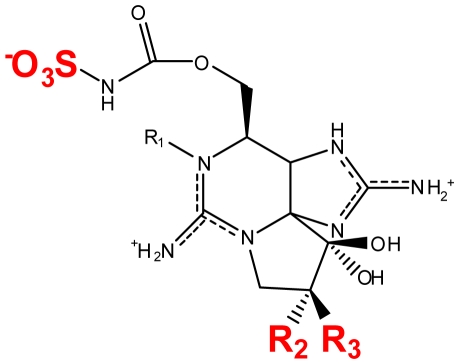	**Di-sulfated**	
	C1-4	<0.01–0.14

Ω Refer to Table 1 for assigned R groups. Moieties highlighted in red differentiate from the structure of STX;

¥ α/β epimeric mixture;

Φ Relative toxicity based on the mouse bioassay results obtained from [95–98];

ω Based on binding affinity to human brain, heart and muscle Na^+^ channels assessed in *Xenopus* oocytes, respectively [89].
